# Microencapsulation of Black Carrot Anthocyanins by Conventional and Three-Nozzle Spray-Drying: Effect on Storage Stability and Bioaccessibility

**DOI:** 10.3390/foods15162811

**Published:** 2026-08-12

**Authors:** Cristina Vergara, María Teresa Pino, Javier Parada, Olga Zamora, Paz Robert, Paula García, María José Farias

**Affiliations:** 1 Instituto de Investigaciones Agropecuarias, INIA La Platina, Santa Rosa 11610, Santiago 8831314, Chile; mtpino@inia.cl (M.T.P.); olga.zamora@inia.cl (O.Z.); cotee.g@gmail.com (M.J.F.); 2 Instituto de Ciencia y Tecnología de los Alimentos (ICYTAL), Facultad de Ciencias Agrarias y Alimentarias, Universidad Austral de Chile, Av. Julio Sarrazin s/n Campus Isla Teja, Valdivia 5090000, Chile; 3 Departamento de Ciencia de los Alimentos y Tecnología Química, Facultad de Ciencias Químicas y Farmacéuticas, Universidad de Chile, Santiago 8380000, Chile; proberts@uchile.cl; 4 Departamento de Nutrición, Facultad de Medicina, Universidad de Chile, Santiago 8380453, Chile; pgarcia@uchile.cl

**Keywords:** anthocyanins, black carrot, microencapsulation, bioaccessibility, in vitro digestion

## Abstract

The black carrot (*Daucus carota* L.) is a source of anthocyanins (AT), especially acylated forms, valued for their color and antioxidant properties, which are linked to the prevention of diseases caused by oxidative stress. However, their low robustness to pH and temperature limits their application. This work aimed to evaluate the effect of spray-drying microencapsulation of black carrot juice (BCJ) on the stability and bioaccessibility of AT. Spray-drying (SD) was used as an encapsulation technique with conventional (two-nozzle) and three-nozzle spray-drying, and maltodextrin (MD) was used as the encapsulating agent; sodium alginate (SA) was used as the outer layer. The optimal SD conditions were 150 °C and a BCJ:MD ratio of 1:2. Two systems were obtained: BCJ-MD and (BCJ-MD)-SA, both with encapsulation efficiencies above ~90%. During storage for 198 days at 60 °C, both systems significantly reduced the degradation constant compared to free juice, following pseudo-first-order kinetics. However, a critical finding was that in vitro bioaccessibility was significantly higher in the conventional BCJ-MD (~88%) compared to the BCJ-MD-SA system (~44%). These results suggest that maltodextrin alone is a more effective strategy for maximizing the in vitro bioaccessibility of black carrot anthocyanins, thereby providing a stable, highly bioaccessible healthy ingredient for the food industry.

## 1. Introduction

Consumers’ changing habits have led to a notable rise in demand for functional meals, which are seen as wholesome. This has also increased interest in replacing synthetic dyes with natural colorants, mainly because synthetic colorants have been restricted by official regulations in the EU and the USA due to their potential adverse effects on human health [1,2].

In this regard, the black carrot (*D. carota* L.) is a potential source of natural colors. It is also a good source of minerals, vitamins, and polyphenols, such as anthocyanins. Its composition includes approximately 88% water, 1% protein, 8% carbohydrate, 0.14% fat, and 2.5% fiber [3,4,5]. The black carrot stands out for its attractive bluish-purple color and high levels of anthocyanins [6,7]. These compounds produce a bright red color under acidic conditions. Black carrots are used as a nutraceutical ingredient in various food matrices, including fruit juices, soft drinks, yogurt, jellies, and confectionery [6,8,9]. Consequently, using black carrot extract as a natural colorant may also provide health benefits and enhance the oxidative stability of foods [6,8]. The main anthocyanins are cyanidin derivatives; among them, 64–77% are acylated anthocyanins. A significant property of cyanidins is their antioxidant activity, which plays an important role in preventing neuronal and cardiovascular diseases, cancer, and diabetes, among others [3,4,6,10,11,12,13,14,15,16].

Black carrots are reported to contain up to 350 mg of anthocyanins per 100 g of fresh weight. For comparison, total anthocyanin concentrations are approximately 113 mg/100 g in blueberries and 48 mg/100 g in raspberries [12]. The structure of anthocyanins is affected by pH, temperature, water activity, exposure to light and oxygen, enzymatic activity, the presence of co-pigments, self-association, metallic ions, ascorbic acid, sugar, and their degradation products [4,15,16,17,18,19]. Stability is an important consideration when using anthocyanins as antioxidants. Foods containing anthocyanins are thermally processed before consumption, and this processing can significantly influence the anthocyanin content of the final product [4,18,19].

The first challenge for anthocyanin (cyanidin)-based ingredients is protecting the actives from environmental conditions. The second challenge is preserving antioxidant capacity and bioaccessibility until they are ingested by consumers. These aspects can be addressed using microencapsulation to protect cyanidins [2,20,21,22] and/or to control cyanidin release at a specific site or time [23].

Encapsulation by spray-drying (SD) has been described as a technique in which an active compound is introduced into or entrapped within a polymeric wall to protect it from environmental conditions, interactions with other food components, and exposure to the gastrointestinal tract, or to control the specific conditions (time and/or site) of active compound release [21]. Among encapsulation technologies, spray-drying is considered one of the most suitable approaches for producing stable food ingredients because it is a continuous, scalable, and cost-effective process that provides high encapsulation efficiencies while preserving heat-sensitive bioactive compounds through rapid water evaporation. In addition, the technology is widely used by the food industry due to its industrial feasibility and compatibility with food-grade wall materials. SD equipment commonly uses a conventional two-way nozzle, which allows spray-drying of a single feed solution and is therefore limited to feed solutions containing compatible materials (conventional SD) [24]. By contrast, using a different nozzle, such as a three-way nozzle, during SD may be a suitable strategy for forming an outer layer coating microparticles, since the solution sprayed by the external peripheral nozzle covers the solution sprayed by the central nozzle during the atomization process, generating an outer layer [25,26,27,28].

Most previous studies on black carrot anthocyanins have focused on conventional spray-drying using single-wall systems, whereas the potential advantages of multilayer microparticles produced by three-fluid nozzle spray-drying for simultaneously improving storage stability and gastrointestinal bioaccessibility remain poorly understood. Various encapsulating agents have been used to encapsulate black carrot anthocyanins by spray-drying (maltodextrins, proteins, and others). Among the biopolymers available, maltodextrins with different dextrose equivalents (DEs) are widely used as encapsulating agents in spray-drying because they have high water solubility and low viscosity, allowing an infeed solution with high solid content; in addition, they have a bland flavor and produce colorless solutions [20]. For outer layers, sodium alginate has been reported as the most used polymer [26,27]. SA is a controlled-release polymer used as an intestinal trigger. It is an anionic biopolymer that imparts high viscosity and low solids content to infeed solutions. Moreover, it is important to determine how digestion affects polyphenols, as this will influence their bioaccessibility [4,13,29]. Accordingly, the potential health-promoting effects of black carrot anthocyanins depend on their processing, stability during storage, and gastrointestinal tract absorption [4,29]. Some research has shown that microencapsulation can increase the bioaccessibility of anthocyanins by up to 20% compared with free extracts or juices. However, the specific impact of multilayer systems formed with a three-fluid nozzle on the stability of black carrot anthocyanins remains to be thoroughly evaluated [2,15,16,29]. To the best of our knowledge, this is the first study to compare conventional two-fluid and three-fluid nozzle spray-drying for the microencapsulation of black carrot anthocyanins under optimized processing conditions

Therefore, the aim of this study was to develop and optimize spray-dried microencapsulation systems for black carrot juice anthocyanins and to compare the effects of conventional and three-nozzle spray-drying on encapsulation efficiency, storage stability, and in vitro bioaccessibility.

## 2. Materials and Methods

### 2.1. Raw Material

Black carrot (*Daucus carota* L.) cultivar ‘Deep Purple’ was grown at the Instituto de Investigaciones Agropecuarias (INIA), Los Tilos Experimental Station, Santiago, Chile, during the 2022 growing season. The crop was harvested 150 days after sowing, when roots reached the optimal harvest index, soluble solids (>9 °Brix), and anthocyanin accumulation, in accordance with the agronomic recommendations of Pino and Vergara [30]. A representative random sample of healthy, uniform roots was collected from the experimental field and washed, and three independent batches of approximately 1 kg of commercially mature black carrot roots were harvested in 2022 for juice preparation. Each batch was processed independently throughout the study.

Encapsulating agents included maltodextrin (Prinal, Santiago, Chile) and sodium alginate (Alginatos Chile S.A., Paine, Chile).

### 2.2. Black Carrot Juice

#### 2.2.1. Preparation of BCJ

For each batch, approximately 1 kg of black carrot was randomly harvested from the experimental field, washed with cold water spray, and ground in a blender (Thermomix, Vorwerk, Germany) to obtain a juice according to Vergara et al. [2]. Briefly, the pH was adjusted to 3 with a citric acid solution to inactivate oxidation, and black carrot juice was then extracted by pressing. The juice was centrifuged, and the supernatant was concentrated in a rotary evaporator R-100 (Büchi, Flawil, Switzerland) at 50 °C until reaching 65 °Brix. BCJ was then stored at −20 °C until analysis.

#### 2.2.2. Characterization of Black Carrot and BCJ

The black carrot (raw material) and BCJ were characterized by moisture, measured with an infrared moisture analyzer (PMB202, ADAM, Oxford, UK), and soluble solids (°Brix), measured with a refractometer (HI 96801, Hanna Instruments, Woonsocket, RI, USA).

AT content: Total anthocyanins were determined by the pH-differential method [31], as described by Lee et al., using spectrophotometry. This method includes measurements at pH 1 and 4.5, with absorbance at 510 and 700 nm. Anthocyanin content was quantified by mixing 200 µL of the sample with 800 µL of buffer at pH 1 or pH 4.5. The mixture was filtered through a 0.45 µm syringe filter, and measurements were taken. Results were expressed as Cyanidin-3-glucoside equivalents per 100 g of fresh weight, using an external calibration curve for Cyanidin-3-glucoside under the same conditions as the samples.

AT profile: The anthocyanin profile and quantification were performed by HPLC-DAD [32]. The ATs were extracted from 0.2 g of BCJ using 50 mL of extraction solution (10% acetic acid or 0.2% trifluoroacetic acid in methanol). All samples were filtered through a 0.45 µm syringe filter prior to injection. Using HPLC equipment with a photodiode-array detector (Jasco interface LC-NetII/ADC 7059-J012A with a diode-array detector MD-4010, quaternary pump PU-4180-LPG, column oven CO-4060, and an autosampler AS-4050 with cooling system TC-4000-1 (Jasco, Tokyo, Japan)) and a C18 column PerkinElmer Universal LC C18 (250 × 4.6 mm; 5 µm) column (Waltham, MA, USA), the samples (20 μL) were injected. The mobile phases, A (0.2% *v*/*v* TFA in water), B (0.2% *v*/*v* TFA in acetonitrile), and C (0.2% *v*/*v* TFA in methanol), were used under the following conditions: initial, 7% B and C; linear change to 40 min to 13% B and 13% C. The column oven temperature was 40 °C. Cyanidins were detected at 520 nm. Quantification was carried out using cyanidin-3-O-glucoside as the standard. All analyses were carried out in triplicate.

Antioxidant activity: Antioxidant activity was determined using the FRAP method, as described by Benzie and Strain [33]. All analyses were carried out in triplicate. Results were expressed as mg of Trolox equivalent per 100 g of fresh weight.

### 2.3. Microencapsulation of BCJ by Spray-Drying

#### 2.3.1. Conventional Spray-Drying

Statistical design: Encapsulation of BCJ with MD (BCJ-MD) was performed using a mini-spray dryer B-290 (Büchi, Flawil, Switzerland) with a conventional two-fluid nozzle. The experimental conditions were set according to a central composite design (12 runs) with a BCJ/MD ratio 1:0.5–1:2 and an inlet air temperature 120–180 °C. The drying conditions were airflow of 600 L/h, atomization pressure of 20 psi, and feeding rates of 0.12 mL/min and 1.2 mL/min for the inner and outer infeeds, respectively. The response variable was encapsulation efficiency (EE) of AT, calculated according to Equation (1). Response surface methodology (RSM) was applied to optimize EE, aiming to maximize it. Data were fitted to a second-order regression model that included linear, quadratic, and cross-product terms for inlet air temperature and the BCJ/MD ratio.

(1) $$\mathrm{EE}\;(\%)=\frac{Total\;AT-surface\;AT}{total\;AT\;experimental}\times 100$$

#### 2.3.2. Three-Fluid Nozzle Spray-Drying

The encapsulation of BCJ with MD was optimized using an experimental design with a two-fluid nozzle (conventional spray-drying). Then, microparticles of BCJ-MD-SA were prepared under optimized BCJ-MD conditions using a three-fluid nozzle (0.7 mm inner core feed diameter and 2.0 mm outer feed/tip orifice) (Technical Data sheet BUCHI. COM). The preparation of the inner and outer infeed and the spray-drying conditions was carried out following the method of Cáceres et al. [26].

Inner infeed: MD was dissolved in distilled water, and then mixed with the AT and homogenized at 20,000 rpm for 2 min using a Polytron PT-2100 (Kinematica AG, Luzern, Switzerland).

Outer infeed: SA was dispersed in distilled water under magnetic stirring for 2 h. The inner:outer infeed ratio was 1:10 to prepare the outer layer, following Cáceres et al. [27].

Spray-drying: To prepare microparticles with an outer layer of BCJ-MD-SA, the inner and outer infeeds were delivered via a mini-spray using a three-fluid nozzle. Drying conditions were airflow of 600 L/h, atomization pressure of 20 psi, feeding rates of 0.12 mL/min and 1.2 mL/min for the inner and outer infeeds, respectively, and an inlet air temperature of 150 °C. Microparticles were stored at −20 °C until analysis.

#### 2.3.3. Characterization of BCJ-MD and BCJ-MD-SA Microparticles

##### Encapsulation Efficiency of AT

The EE of AT was calculated according to Equation (1).

Experimental total anthocyanin determination: Microparticles (200 mg) were dispersed in 2 mL of methanol:acetic acid:water (50:8:42 *v*/*v*/*v*) and vortexed for 1 min. They were then ultrasonicated twice for 10 min and finally centrifuged at 11,200× *g* for 5 min [2]. The anthocyanin content was determined by the pH-differential method and HPLC-DAD.

Superficial anthocyanin determination: Microparticles (100 mg) were dispersed in 2 mL of ethanol:methanol (1:1), vortexed for 1 min, and then centrifuged at 11,200× *g* for 5 min [2]. Anthocyanin content was determined by the pH-differential method and by HPLC-DAD.

##### Moisture Content and Water Activity

The moisture content was measured with an infrared moisture analyzer (PMB202, ADAM, Oxford, UK). Water activity (aw) was measured using the dewpoint method (Hygrolab 2, Rotronic, Huntington, USA; 20 ± 0.3 °C).

##### Morphology

The outer structures of the microparticles were examined by scanning electron microscopy (SEM). The powder was coated with gold/palladium using a Varian PS 10E vacuum evaporator and then analyzed with a LEO 1420VP SEM (LEO Electron Microscopy Ltd., Cambridge, UK) operating at 20 kV. The images were then collected digitally using EDS 7424 software version 4.1.13.2167 (Oxford Instruments, Oxford, UK).

### 2.4. The Storage-Accelerated Stability of BCJ Microparticles

The BCJ microparticles obtained under optimal conditions by spray-drying BCJ-MD and BCJ-MD-SA (100 mg) were transferred to clear glass vials (16 × 100 mm) and stored at 60 ± 1 °C in a forced-air oven (UFE 500, Memmert, Schwabach, Germany) with controlled temperature and in the absence of light (in triplicate). The progression of AT degradation was monitored by collecting seven samples at specific time intervals. Anthocyanin contents were quantified by HPLC-DAD, and the anthocyanin degradation rate constant was determined. To determine the kinetic degradation of anthocyanin, the study was conducted over a 6-month period.

Kinetic analysis. The data were best fit by a first-order kinetic model according to Equation (2):

LnC = LnC_0_ − *kt* (2)

where C_0_ is the initial concentration of anthocyanin (mg/g), C is the anthocyanin concentration at time *t*, *k* is the anthocyanins degradation rate constant, and *t* is the storage time. The degradation rate constants (k) and correlation coefficient were obtained from the slope of a plot of the natural log of the percentage retention of anthocyanin versus time for first-order kinetics at each studied temperature (60 °C).

### 2.5. In Vitro Gastrointestinal Digestion

The in vitro gastrointestinal digestion was conducted using a modified static model adapted from Aravena et al. [34], which aligns with the general three-stage sequence (oral, gastric, and intestinal) of the INFOGEST consensus guidelines [35]. Key operational modifications were implemented regarding enzyme dosage and fluid ratios. Unlike the standardized INFOGEST 2.0 protocol—which requires pre-adjusting enzyme additions strictly based on assayed specific enzymatic activity (U/mL or U/g) and maintaining a 1:1 food-to-fluid ratio—enzymes in this study were introduced at fixed weight/volume concentrations (α-amylase: 60 U/mL in buffer; pepsin: 25 mg/mL; pancreatin: 2 g/L; bile salts: 12 g/L in $0.1\;M\;{\mathrm{NaHCO}}_{3}$), tailored for encapsulated microparticle matrices [34]. Digestive enzymes and bile salts were purchased from Sigma–Aldrich Co. (St. Louis, MO, USA): α-amylase from human saliva 300–1000 U mg^−1^ protein (A1031-5KU); pepsin from porcine gastric mucosa ≥ 250 U mg^−1^ solid (P7000-100G); pancreatin 4 USP (P1750-100G); and bile extract porcine (B8631-100G).

To evaluate recovery efficiencies, the digesta underwent sequential separation steps. The intestinal digesta was acidified to pH 3.0 to stop enzymatic activity and stabilize anthocyanins, followed by low-speed centrifugation (12,000× g for 10 min) to precipitate insoluble remnants. The resulting liquid supernatant was further clarified at 12,000× g to isolate the bioaccessible fraction (${\mathrm{AT}}_{d}$). Overall efficiency and bioaccessibility (BA %) were determined as the proportion of total initial anthocyanins (${\mathrm{AT}}_{m}$) released into the final liquid volume ($V_{T}$), as detailed in Equation (3).

In brief, the digestive process includes the following stages:

First, for oral digestion, artificial saliva (9 mL) was added to each flask containing a sample (about 0.5 g of microparticles). The artificial saliva contained 14.4 mM sodium bicarbonate, 21.1 mM potassium chloride, 1.59 mM calcium chloride, and 0.2 mM magnesium chloride. The pH was adjusted to 7 with 1 M HCl. On the day of analysis, 60 α-amylase units per milliliter of buffer were added. Samples were incubated at 37 °C at 185 rpm in a shaking water bath (Jeio Tech, model BS-06, Daejeon, Republic of Korea).

For gastric digestion, after the oral stage, the pH of the samples was adjusted to 2.0 with 1 M HCl, and 36 mL of a pepsin solution (25 mg/mL in 0.02 M HCl) was added. Samples were incubated for 2 h at 37 °C with stirring.

Finally, the gut digestion stage was performed by first adjusting the samples’ pH to 6.0 with NaHCO_3_ (1 M), and then adding 0.25 mL of an artificial gut solution per mL of sample, containing pancreatin (2 g/L) and bile salts (12 g/L) dissolved in NaHCO_3_ (0.1 M). The samples were incubated for 2 h at 37 °C with shaking at 45 rpm. Each digestion product was transferred to 50 mL Falcon tubes, and the pH was adjusted to 3 [34]. The tubes were then centrifuged for 10 min at 11,200× *g* to recover the liquid fraction. Next, the liquid digestion product was centrifuged at 11,200× *g* before anthocyanin analysis. Anthocyanin bioaccessibility was calculated according to Equation (3) and AT content was determined by the pH-differential method and by HPLC-DAD (according to Section 2.2.2).

(3) $$Bioaccessibility\;(BA)\left[\%\right]=\frac{{AT}_{d}\times V_{T}}{{AT}_{m}\times G}\times 100$$

where ${AT}_{d}$ is the anthocyanin in the liquid phase after digestion, representing the fraction released from the microparticle matrix; *V_T_* is the final volume after digestion; ${AT}_{m}$ is the anthocyanin in the microparticles before digestion; and *G* is the amount of sample analyzed.

### 2.6. Statistical Analysis

Differences among particle systems for each parameter were analyzed using ANOVA and Tukey’s test. Linear regression with 95% confidence limits was employed to establish the reaction order and the cyanidin release constant during studies of particle stability, both during storage and under simulated digestion. To determine statistical differences in release rates across temperature and release medium, ANOVA was carried out, and all statistical analyses were performed with Statgraphics Plus 7.0

## 3. Results

### 3.1. Characterization of Black Carrot and BCJ

**Figure 1:**
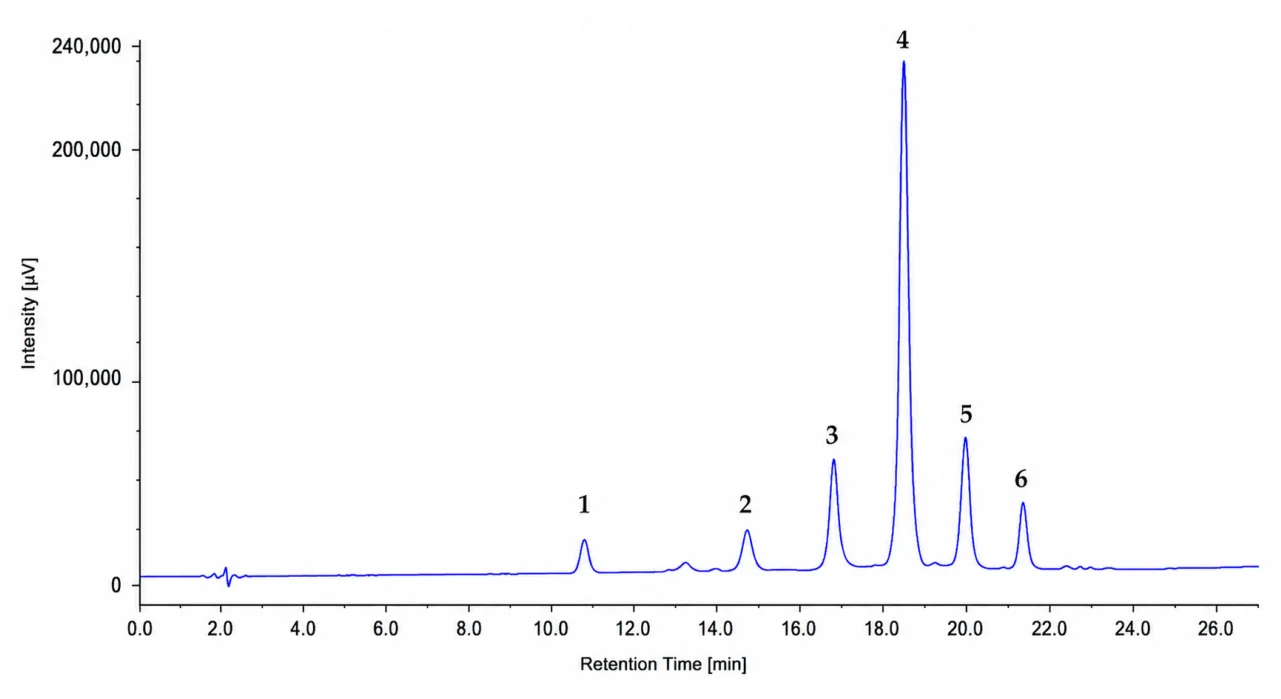
HPLC chromatogram of the anthocyanins from black carrot (520 nm). Peak 1: Cyanidin-3-xylosyl-glucosyl-galactoside; peak 2: Cyanidin-3-xylosyl-galactoside; peak 3: Cyanidin-3-xylosyl(sinapoylglucosyl)galactoside; peak 4: Cyanidin-3-xylosyl(feruloylglucosyl)galactoside; peak 5: Cyanidin-3-xylosyl(coumaroylglucosyl)galactoside; peak 6: Peonidin-3-xylosyl-galactoside.

The physical and chemical characteristics of black carrot (raw material) and BCJ are presented in Table 1, which also lists the concentrations of individual anthocyanins. Figure 1 shows the chromatogram of black carrot.

The HPLC profile of AT from black carrot showed cyanidin derivatives, including non-acylated and acylated forms with hydroxycinnamic acids (sinapic, ferulic, and coumaric acid) [36,37,38]. The main peak identified by HPLC corresponds to Cyanidin-3-xylosyl(feruloylglucosyl)galactoside, accounting for approximately 83% of the total AT, as was reported by other authors [37,38]. Cyanidin-3-xylosyl-glucosyl-galactoside and Cyanidin-3-xylosylgalactoside correspond to peaks 1 and 2, respectively (non-acylated cyanidin derivatives), and acylated anthocyanins correspond to peaks 3, 4, and 5. The non-acylated anthocyanins are 5.2%, while the acylated anthocyanins are 94.8% in raw black carrot (see Table 1 and Figure 1).

### 3.2. Optimization of Microencapsulation of BCJ

Table 2 shows the experimental runs and their response variable (EE of AT) for the SD microencapsulation of BCJ using MD as encapsulating agent. The EE of AT ranged from 50% to 98%. According to the ANOVA, EE was significantly affected by the linear and quadratic effects of both inlet air temperature and the BCJ:MD ratio (*p* < 0.05).

The equation describing the effect of the independent variables on the EE of AT is the following:

EE (%) = −235.714 + 50.8446 × X_2_ + 3.57029 × X_1_ − 9.04638 × X_2_^2^ − 0.0255556 × X_1_ × X_2_ − 0.0114596 × X_1_^2^ (4)

**Figure 2:**
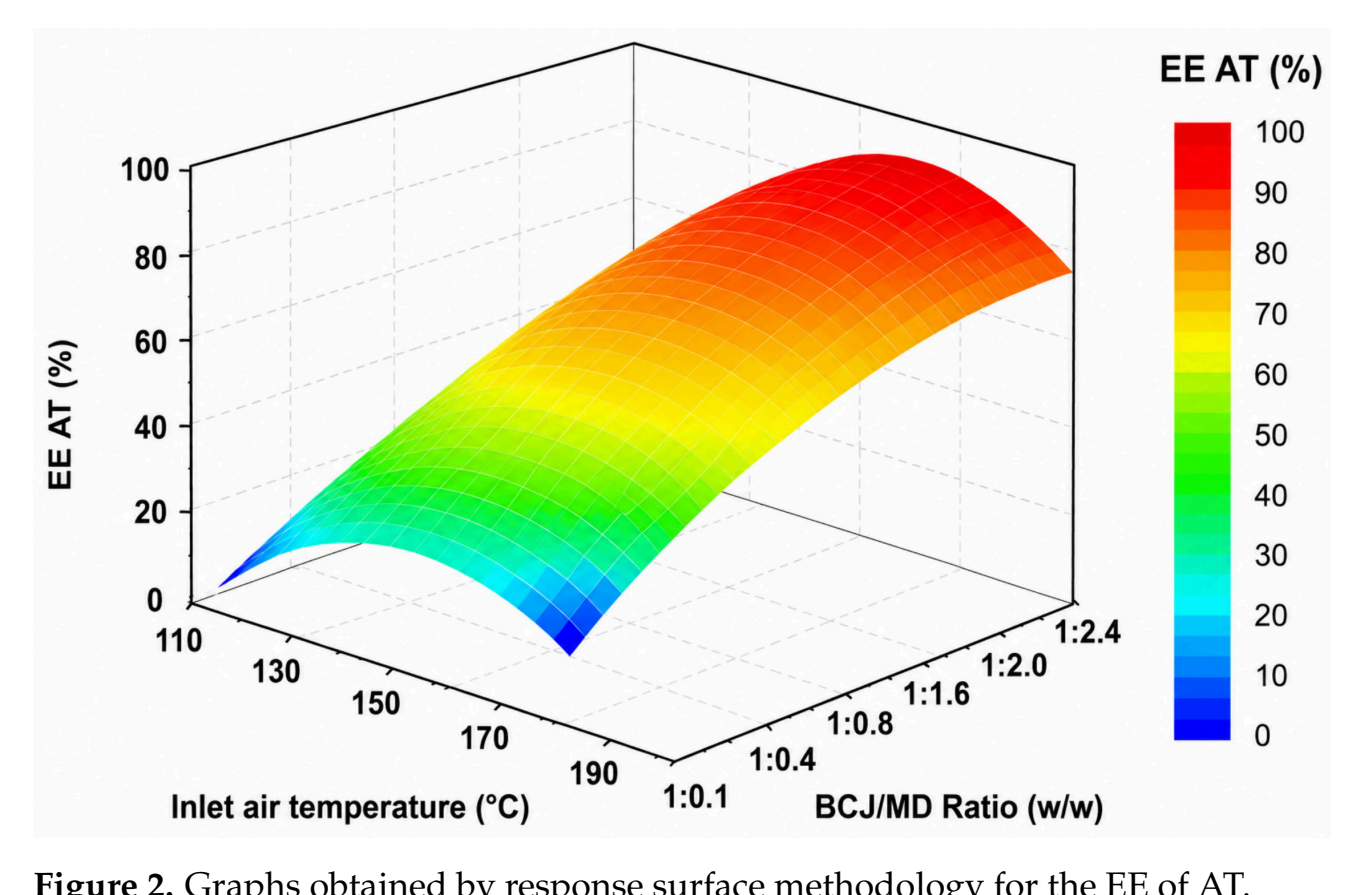
Graphs obtained by response surface methodology for the EE of AT.

The response surface methodology plot is shown in Figure 2. When the BCJ:MD ratio increases and the temperature is intermediate within the studied range, the EE of AT increases (red zone in the plot). This quadratic model explained over 98% of the variability (R^2^ = 98.6% and R^2^ adjusted = 97.3%). The lack of fit was not significant (*p*-value = 0.0697).

The optimal SD conditions were 150 °C (inlet air temperature) with a BCJ:MD ratio of 1:2. These conditions were used to produce microparticles under optimal conditions for conventional SD (BCJ-MD) and three-nozzle SD (BCJ-MD-SA).

### 3.3. Characterization of the BCJ-MD and BCJ-MD-SA Systems Obtained Under Optimal Conditions

Table 3 shows the physical and chemical characteristics of the BCJ-MD and BCJ-MD-SA systems obtained under optimal conditions. It is possible to indicate that the micro-particles obtained under optimal conditions are comparable since there are no significant differences in their water activity or in AT content.

**Figure 3:**
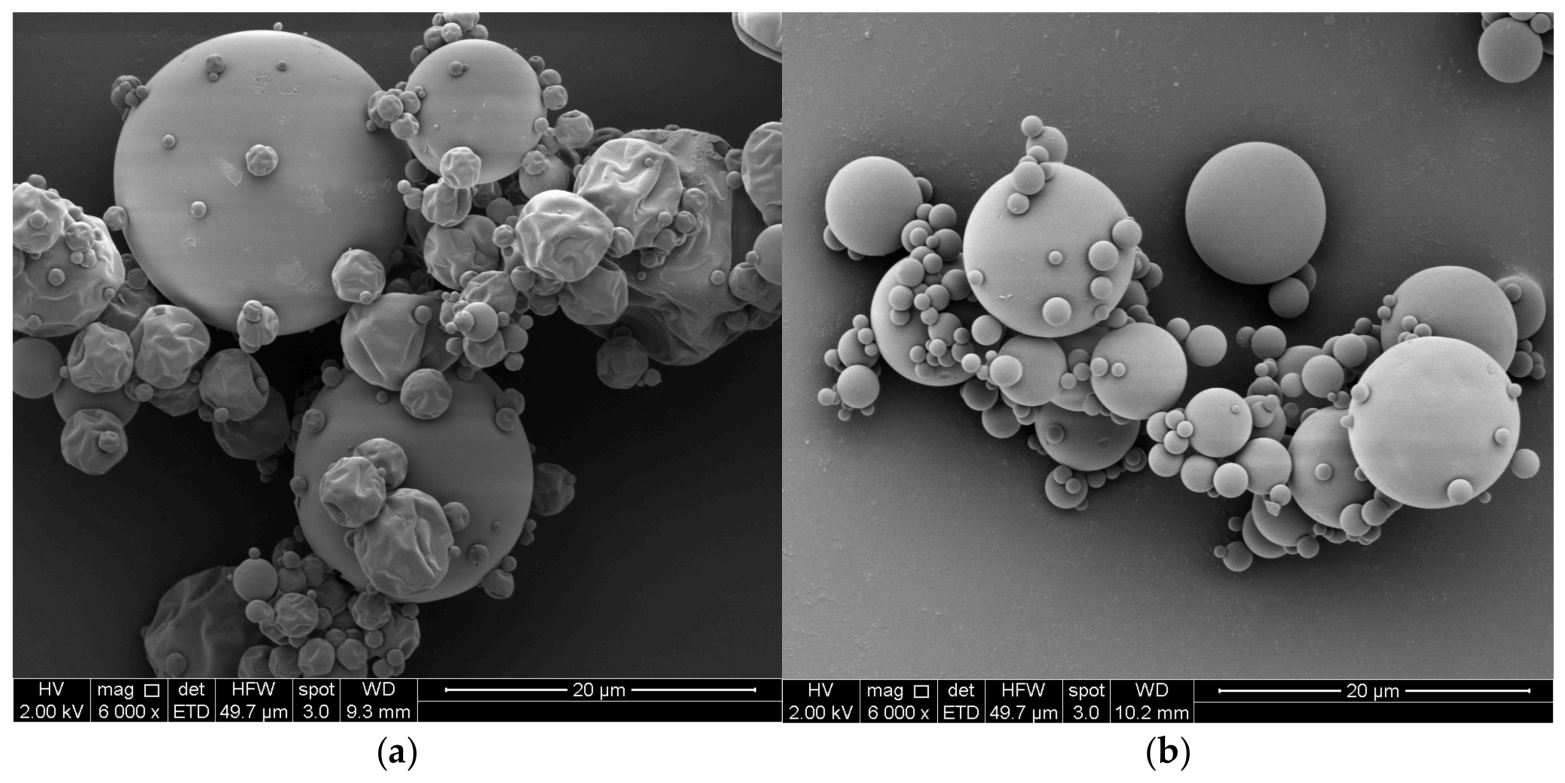
Scanning electron microscopy (SEM) photographs of (**a**) BCJ-MD and (**b**) BCJ-MD-SA.

Figure 3a,b show SEM images of BCJ-MD and BCJ-MD-SA microparticles produced under optimal conditions by conventional spray-drying and using a three-fluid nozzle, respectively.

### 3.4. Stability of BCJ, BCJ-MD, and BCJ-MD-SA Microparticles During Storage at 60 °C

**Figure 4:**
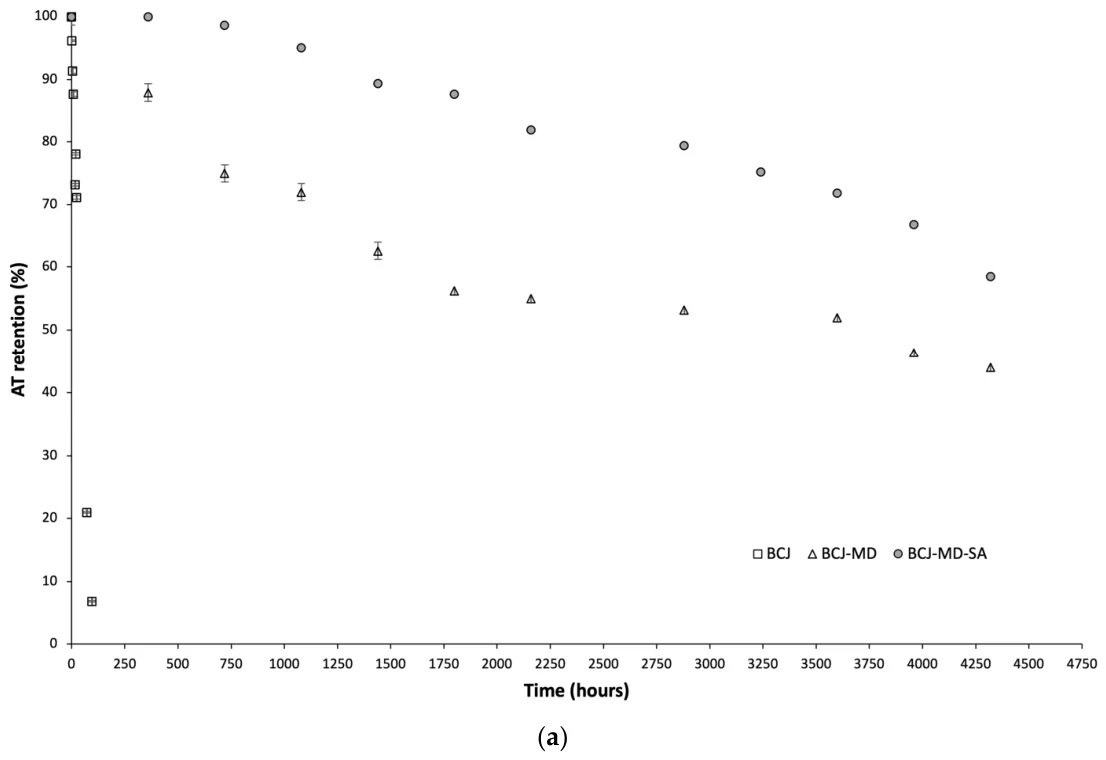

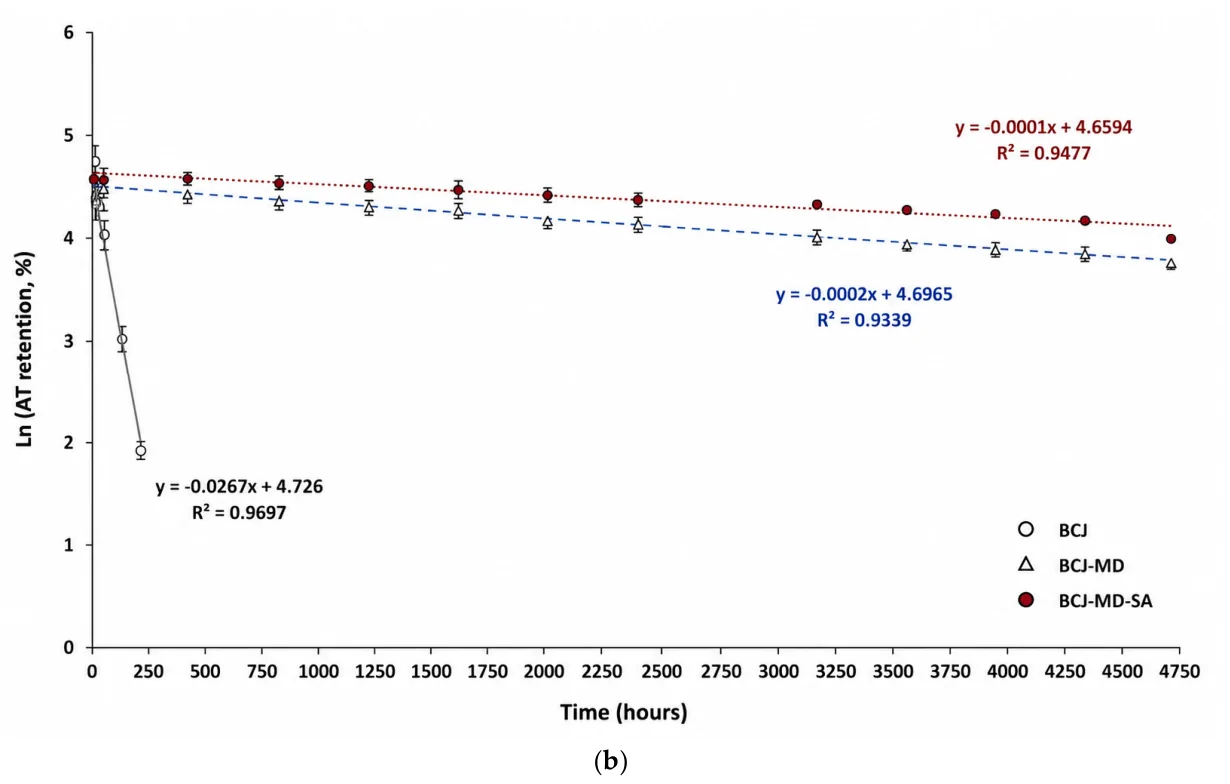
(**a**) Graph of the evolution of AT retention over time (hours) and (**b**) the degradation rate constant of AT at 60 °C.

Figure 4a shows the evolution of AT retention over time (hours). To evaluate the effects of storage temperature, the initial total AT content of encapsulated black carrot anthocyanins at 0 h was set to 100%. The stability and retention of encapsulated black carrot anthocyanins were monitored for 4750 h (198 days) using AT non-encapsulated (BCJ), conventional SD (BCJ-MD), and three-fluid nozzle SD (BCJ-MD-SA).

Figure 4b shows the fit to first-order kinetics (k_obs_ and r^2^) for BCJ (non-encapsulated), BCJ-MD, and BCJ-MD-SA microparticles, respectively. The AT retention data were best fit by a first-order kinetic model (Equation (2)). The degradation rate constants (k_obs_) and correlation coefficient (r^2^) were obtained from the slope of a plot of the natural log of the percentage retention of anthocyanin versus time at 60 °C.

All systems follow first-order kinetics that depend on the concentration of the anthocyanin compound. The AT degradation rate constant (k_obs_) was 26.7 × 10^3^ h^−1^ (r^2^ 96.9%) for free AT (non-encapsulated) BCJ, and 2.0 × 10^4^ h^−1^ (r^2^ 96.7%) and 1.0 × 10^4^ h^−1^ (r^2^ 94.8%) for BCJ-MD and BCJ-MD-SA microparticles, respectively.

### 3.5. In Vitro Bioaccessibility of Anthocyanins from BCJ, BCJ-MD, and BCJ-MD-SA Microparticles

Table 4 presents the final bioaccessibility (BA) (%) of AT after in vitro digestion of BCJ (non-encapsulated) and of BCJ-MD and BCJ-MD-SA microparticles. Table 4 shows BA for the total AT (calculated by the pH-differential method) and BA for individual AT, determined by HPLC-DAD. The BCJ-MD microparticles reach the highest final BA of AT, and for individual ATs. The BA values exceeding 100% observed for Cyanidin-3-xylosyl-glucosyl-galactoside should not be interpreted as a net increase in anthocyanin content. Instead, they likely result from the partial deacylation and hydrolysis of acylated anthocyanins during simulated gastrointestinal digestion, leading to the formation of this non-acylated derivative. Consequently, the concentration of this individual anthocyanin detected by HPLC after digestion was higher than its initial concentration. Similar transformations of acylated anthocyanins under digestive conditions have been previously reported [16].

## 4. Discussions

### 4.1. Characterization of Black Carrot and BCJ

Moisture levels, soluble solids, antioxidant activity, and AT content are comparable with the reported ranges for black carrots [8,36,37] (see Table 1). The AT content in black carrot is consistent with the literature, ranging from 1000 to 1750 mg/kg fresh weight [15,36,37]. The BCJ reached 65 °Brix, and its AT content and antioxidant activity were significantly higher than those of raw black carrot due to the juice-concentration process. These parameters fall within the typical range for a concentrated juice produced by evaporating a water extract.

The HPLC profile of anthocyanins from black carrots shows a predominance of acylated forms (94.8%), with Cyanidin-3-xylosyl(feruloylglucosyl)galactoside being the majority component (~83%). Similar results have already been reported [10,36,38,39]. According to the literature, acylated anthocyanins from black carrot are up to six times more stable and resistant to hydration and pH changes than non-acylated anthocyanins [6].

### 4.2. Microencapsulation of BCJ

This study employed a central composite design, optimized using RSM, to identify the optimal conditions for microencapsulating BCJ with MD via spray-drying. The independent variables for EE of AT were the inlet air temperature (a process variable) and the BCJ:MD ratio (a formulation variable). RSM was applied to maximize the response variable, that is, to obtain EE values for carrot and blackberry anthocyanins close to 100%.

The EE of AT ranged from 50% to 98%. The ANOVA for the EE of anthocyanins showed that the EE of ATs was significantly affected by the linear and quadratic forms of the inlet air temperature and the BCJ:MD ratio (*p* < 0.05). This model explained over 98% of the variability (R^2^ = 98.6% and R^2^ adjusted = 97.3%), with residuals below 4.0. The lack of fit was not significant (see Table 2), indicating that the mathematical model fits the experimental data well.

For the BCJ:MD ratio, the EE of AT rose with increasing MD content, likely due to rapid dry-crust formation on droplet surfaces. In contrast, inlet air temperature exerted only a minor effect on the EE of AT [2,40].

The optimal spray-drying conditions were 150 °C with a BCJ:MD ratio of 1:2 for both conventional SD processes.

The same spray-drying conditions were applied to the three-nozzle SD processes, applying SA to the outer layer.

### 4.3. Characterization of the BCJ-MD and BCJ-MD-SA Systems Obtained Under Optimal Conditions

The moisture, a_w_, and AT content were within the range reported for polyphenols or anthocyanin microparticles obtained by spray-drying with MD [22,25,29,31].

The EE of a microparticle depends on achieving high retention of the core material within the encapsulation wall material and minimizing its presence on the microcapsule surface. The EE of microparticles produced with MD and MD–SA is shown in Table 3. The EE for all treatments exceeds 90%, indicating that the black carrot anthocyanin encapsulation process was successful. The EE in both systems (BCJ-MD and BCJ-MD-SA) is higher than previously reported values for purple potato juice (86%) [2] and maqui juice (74–85%) using maltodextrin (MD) [41]. This high performance suggests strong hydrogen-bonding interactions between the hydroxyl groups of the AT and the MD matrix [2,22,24,41]. However, recent studies indicate that EE can be further optimized through polymer synergy. Thus, combining MD with sericin proteins [15] or soy isolates has been shown to improve not only EE but also the total recovery of AT after drying by forming a denser protective crust that limits diffusion of the core to the surface. In this study, adding SA via a three-fluid nozzle maintained a high EE (>93%), comparable to the complex systems of gum Arabic and inulin (97.8%) used in myrtle berries [42].

The external morphology observed by SEM images of BCJ-MD and BCJ-MD-SA was produced under optimal conditions; the particles are spherical, with irregular surfaces that tend to agglomerate. The indentations observed on particles during spray-drying result from particle shrinkage, which can occur at both high and low inlet air temperatures. At higher temperatures, rapid water evaporation and increased internal pressure cause shrinkage, whereas at lower temperatures, slower water diffusion leads to longer shrinkage times [43]. Similar external morphologies have been observed in particles of anthocyanin pigment encapsulated by conventional SD with MD, including those from black carrot [15,43], maqui juice [41], and purple potato juice [2].

### 4.4. Stability of BCJ Microparticles During Storage

All systems follow first-order kinetics that depend on the concentration of the anthocyanin compound, driven by oxidation, cleavage, or heating-induced oxidation [44]. In both graphs, the protective effect of microencapsulation is evident. Both microparticle systems significantly reduced the AT degradation rate constant (26.7 × 10^3^ h^−1^) compared with BCJ (26.7 × 10^3^ h^−1^). No significant differences in the degradation rate constants of anthocyanins were observed between systems with and without an outer layer, suggesting that MD is the primary stabilizing factor via hydrogen-bond interactions. The same order has been reported for AT degradation in microparticle systems from black carrot extract [43], maqui juice [41], and purple potato juice [2], where microencapsulation techniques significantly improved the stability of AT and phenolic compounds. Encapsulation enhanced AT stability through MD–AT interactions, minimizing damage to active AT caused by adverse storage conditions and thereby prolonging shelf life while maintaining AT retention and color.

The enhanced storage stability of anthocyanins observed in the encapsulated systems can be attributed to several complementary protective mechanisms provided by the encapsulating matrix. First, MD and the outer SA layer act as physical barriers that reduce the penetration of oxygen, moisture, and light, thereby limiting oxidation and other degradation reactions during storage [45,46]. In addition, intermolecular interactions, particularly hydrogen bonding between anthocyanins and the hydroxyl groups of MD, may further stabilize the encapsulated compounds by reducing their susceptibility to chemical degradation [2,22,24,41,47]. The low water activity of the spray-dried powders also contributes to improved stability by reducing the rate of degradation reactions [48]. These complementary mechanisms explain the significantly greater stability of encapsulated anthocyanins compared with non-encapsulated BCJ during storage, particularly for the BCJ-MD-SA system, where the additional alginate outer layer provided an extra diffusion barrier against environmental factors [45,47].

### 4.5. In Vitro Bioaccessibility of Anthocyanins

Bioaccessibility is the amount of a compound released from the matrix following simulated in vitro digestion (oral, gastric, and intestinal phases) [44]. Microencapsulation of black carrot anthocyanins with MD effectively protected them against simulated gastric conditions, minimizing chemical degradation in the gut environment (see Table 4). The final BA of total AT was higher for BCJ-MD (~88%) than for BCJ-MD-SA (~44%) and for BCJ (non-encapsulated) (~57%). On the other hand, encapsulation appears to protect the AT during digestion against environmental conditions (especially pH) when MD is used as the encapsulating agent. The final BA of the BCJ and BCJ-MD systems was higher than that reported by Vergara et al. [2] for free and encapsulated purple potato juice and by Fredes et al. (2018) [41] for free and encapsulated maqui juice.

A critical finding of this study was the marked difference in anthocyanin bioaccessibility among the evaluated systems. The significantly lower bioaccessibility observed for non-encapsulated black carrot juice (BCJ, 57%) compared with the encapsulated systems demonstrates the protective effect of spray-drying microencapsulation during gastrointestinal digestion. In non-encapsulated BCJ, anthocyanins are directly exposed to pH changes, digestive enzymes, oxygen, and other components of the gastrointestinal environment, making them more susceptible to degradation and structural transformation. In contrast, microencapsulation provided a protective matrix that reduced the direct exposure of anthocyanins to these adverse conditions, preserving a greater proportion of the compounds until they reached the intestinal phase. This protective effect was particularly evident in BCJ-MD microparticles, which exhibited the highest bioaccessibility (88.4%), indicating that maltodextrin efficiently protected the anthocyanins during the oral and gastric phases while allowing their subsequent release during intestinal digestion. Conversely, BCJ-MD-SA microparticles showed a significantly lower bioaccessibility (44.1%). This reduction is unlikely to be associated with greater anthocyanin degradation but rather with the physicochemical behavior of sodium alginate during intestinal digestion. Under intestinal conditions, sodium alginate absorbs water and forms a hydrated gel network that acts as a diffusion barrier, slowing the migration of anthocyanins from the microparticle core into the surrounding medium. Consequently, although the alginate outer layer enhanced anthocyanin stability during storage by providing an additional barrier against oxygen and moisture diffusion, it also restricted the release of encapsulated anthocyanins during digestion, resulting in a lower measured in vitro bioaccessibility. Similar diffusion-controlled release behavior has been widely reported for alginate-based delivery systems, in which gel swelling and the formation of a three-dimensional polymeric network delay the diffusion of encapsulated bioactive compounds and promote sustained release under intestinal conditions [49,50,51]. Interestingly, previous studies have reported anthocyanin bioaccessibility values exceeding 91% for black carrot anthocyanins encapsulated in maltodextrin–protein matrices [15], suggesting that protein-based wall materials may facilitate a more efficient gastrointestinal release than sodium alginate while still providing effective protection during digestion.

When examining the achieved BA of individual anthocyanins, it is evident that for non-encapsulated BCJ and BCJ-MD microparticles, the final BA of individual non-acylated forms can reach 100%. This is explained by deacetylation during ingestion and intestinal transit of acetylated anthocyanins, which converts them into their non-acylated forms (see Table 4, Cyanidin-3-xylosyl-glucosyl-galactoside and Cyanidin-3-xylosyl-galactoside). This aligns with observations by Carrillo et al. [16], who report that intestinal conditions can cause the cleavage of Cyanidin-3-xylosyl(feruloylglucosyl)galactoside (the main anthocyanin of black carrot) to Cyanidin-3-xylosyl-glucosyl-galactoside. Additionally, HPLC data confirmed deacetylation during intestinal digestion; the complex acylated forms of black carrot degraded into simpler non-acylated cyanidins due to neutral pH and enzymatic activity. This phenomenon is crucial because although the BA of the complex forms decreases, the availability of simpler forms may influence subsequent colonic absorption. The determining factor was pH and its impact on bioaccessibility. While anthocyanins are stable under acidic conditions, a shift to a neutral pH in the intestine promotes their transformation or degradation.

From a practical perspective, the BCJ-MD microparticles developed in this study have significant potential as clean-label natural colorants and functional food ingredients. Their high anthocyanin retention during storage and enhanced in vitro bioaccessibility make them suitable for use in acidic beverages, yogurt and fermented dairy products, fruit preparations, confectionery, bakery, powdered beverages, and other dry food formulations. Furthermore, spray-drying, a well-established and scalable industrial technology, facilitates the commercial production of these ingredients. Therefore, the optimized BCJ-MD system represents a promising strategy to replace synthetic colorants while simultaneously increasing the delivery of health-promoting anthocyanins in food products.

## 5. Conclusions

Microencapsulation by spray-drying with maltodextrin as the encapsulating agent is a highly effective technique for stabilizing anthocyanins from black carrot. Adding an outer sodium alginate layer via a three-fluid nozzle did not confer additional thermal stability and drastically reduced the bioaccessibility of the active compounds. The in vitro digestion study showed that MD has the potential to improve microcapsule stability during passage through simulated oral, gastric, and intestinal conditions, thereby leading to high bioaccessibility. Therefore, a better strategy is to use MD alone to impart stability to the AT for formulating healthy ingredients based on black carrots.

## Figures and Tables

**Table 1:** Physical and chemical characterization of black carrot and black carrot juice (BCJ).

Analyze		Black Carrot	BCJ
Moisture content (%)		90 ± 5.1 ^a^	35.0 ± 0.4 ^b^
Soluble solids (°Brix at 20 °C)		9.2 ± 0.2 ^b^	65.0 ± 0.5 ^a^
Antioxidant capacity (FRAP) (mg TE/100 g)		762.1 ± 43.9 ^b^	2123.3 ± 67.5 ^a^
Total anthocyanins (mg Cy-3-glu E/100 g)		165.9 ± 8.5 ^b^	213.8 ± 8.7 ^a^
Total anthocyanins HPLC (mg/100 g)	Cyanidin-3-xylosyl-glucosyl-galactoside	1.3 ± 0.1 ^b^	8.8 ± 0.1 ^a^
	Cyanidin-3-xylosyl-galactoside	2.2 ± 0.2 ^b^	19.6 ± 0.2 ^a^
	Cyanidin-3-xylosyl(sinapoylglucosyl)galactoside	2.0 ± 0.1 ^b^	56.2 ± 5.2 ^a^
	Cyanidin-3-xylosyl(feruloylglucosyl)galactoside	55.3 ± 3.0 ^b^	457.2 ± 10.0 ^a^
	Cyanidin-3-xylosyl(coumaroylglucosyl)galactoside	5.7 ± 0.2 ^b^	56.8 ± 5.0 ^a^
	Peonidin-3-xylosyl-galactoside	0.5 ± 0.1 ^b^	6.3 ± 1.1 ^a^

BCJ: black carrot juice concentrate; Cy-3-glu E/100 g: Cyanidin-3-glucoside equivalent; TE: Trolox equivalent. Different letters indicate statistically significant differences between systems for the Tukey multiple-range test (*p* < 0.05).

**Table 2:** Encapsulation efficiency of anthocyanins (EE) for the BCJ microparticles by spray-drying according to the central composite design and ANOVA.

Run	Inlet Air Temperature (°C) (X_1_)	BCJ:MD Ratio (X_2_)	EE (%)
1	120	1:0.5	50.0 ± 0.5
2	120	1:2.0	89.0 ± 0.7
3	180	1:0.5	57.9 ± 0.2
4	180	1:2.0	94.6 ± 0.1
5	150	1:0.34	55.8 ± 0.1
6	150	1:2.15	98.0 ± 0.2
7	114	1:1.25	66.2 ± 0.3
8	186	1:1.25	72.3 ± 0.1
9	150	1:1.25	88.5 ± 0.6
10	150	1:1.25	87.2 ± 0.4
11	150	1:1.25	85.4 ± 0.7
12	150	1:1.25	87.8 ± 0.2
Source	*p*-value		
X_1_: Inlet air temperature	0.0094 *		
X_2_: BCJ:MD ratio	0.0000 *		
X_1_ X_1_	0.0005 *		
X_1_ X_2_	0.4501		
X_2_ X_2_	0.0042 *		
Lack of fit	0.0697		
R^2^ (%)	98.5		
R^2^ adjusted (%)	97.3		

BCJ: black carrot juice concentrate; MD: maltodextrin; EE: encapsulation efficiency. * indicate statistically significant differences between systems for the Tukey multiple-range test (*p* < 0.05).

**Table 3:** Physical and chemical characteristics of the BCJ-MD and BCJ-MD-SA systems obtained under optimal conditions.

Analyze/Microparticles		BCJ-MD	BCJ-MD-SA
Moisture (%)		3.8 ± 0.3 ^b^	5.3 ± 0.1 ^a^
Water activity		0.29 ± 0.02 ^a^	0.26 ± 0.04 ^a^
Total AT (mg C-3-G/g)		1.95 ± 0.04 ^a^	1.54 ± 0.19 ^a^
EE (%) * Total anthocyanins		90.9 ± 0.4 ^a^	93.2 ± 0.1 ^a^
EE (%) **	Cyanidin-3-xylosyl-glucosyl-galactoside	96.1 ± 0.3 ^a^	94.7 ± 0.1 ^a^
	Cyanidin-3-xylosyl-galactoside	95.4 ± 0.05 ^a^	93.5 ± 0.1 ^a^
	Cyanidin-3-xylosyl(sinapoylglucosyl)galactoside	98.7 ± 0.2 ^a^	96.1 ± 0.1 ^a^
	Cyanidin-3-xylosyl(feruloylglucosyl)galactoside	96.0 ± 0.04 ^a^	94.8 ± 0.01 ^a^
	Cyanidin-3-xylosyl(coumaroylglucosyl)galactoside	96.3 ± 0.1 ^a^	94.9 ± 0.1 ^a^

BCJ: black carrot juice concentrate; MD: maltodextrin; SA: sodium alginate; * EE of total anthocyanins determined by pH-differential method; C-3-G: Cyanidin-3-glucoside; ** EE of individual AT determined by HPLC-DAD. Different letters indicate statistically significant differences between systems in the Tukey multiple-range test (*p* < 0.05).

**Table 4:** Anthocyanins total and individual content in black carrot juice and microparticle powders, and the bioaccessibility of black carrot anthocyanins after an in vitro digestion model.

Anthocyanins	BCJ		BCJ-MD		BCJ-MD-SA	
	mg/g	BA%	mg/g	BA%	mg/g	BA%
Cyanidin-3-xylosyl-glucosyl-galactoside	0.04 ± 0.001	100 ± 0.0 ^b^	0.04 ± 0.001	126.4 ± 2.6 ^a^	0.03 ± 0.001	36.7 ± 0.8 ^c^
Cyanidin-3-xylosyl-galactoside	0.03 ± 0.001	32.3 ± 4.3 ^b^	0.04 ± 0.001	102.1 ± 2.7 ^a^	0.02 ± 0.001	24.0 ± 0.7 ^c^
Cyanidin-3-xylosyl(sinapoylglucosyl)galactoside	0.11 ± 0.002	46.9 ± 3.9 ^b^	0.12 ± 0.003	89.8 ± 2.8 ^a^	0.10 ± 0.002	48.4 ± 1.8 ^b^
Cyanidin-3-xylosyl(feruloylglucosyl)galactoside	1.09 ± 0.03	53.0 ± 2.8 ^b^	0.88 ± 0.008	67.2 ± 0.3 ^a^	0.81 ± 0.006	41.6 ± 0.0 ^c^
Cyanidin-3-xylosyl(coumaroylglucosyl)galactoside	0.13 ± 0.002	55.5 ± 3.6 ^a^	0.11 ± 0.009	58.2 ± 1.1 ^a^	0.10 ± 0.007	34.2 ± 0.1 ^b^
Total AT by HPLC	1.4 ± 0.33	57.2 ± 3.8 ^b^	1.19 ± 0.02	88.43 ± 3.8 ^a^	1.06 ± 0.001	44.1 ± 1.8 ^c^

BCJ: black carrot juice concentrate; MD: maltodextrin; SA: sodium alginate; AT determined by HPLC-DAD. Different letters indicate statistically significant differences between systems in the Tukey multiple-range test (*p* < 0.05).

## Data Availability

The original contributions presented in this study are included in the article. Further inquiries can be directed to the corresponding authors.
